# An Undiagnosed Ventricular Septal Rupture Presenting as New Onset Heart Failure: A Rare Complication of an Anterior Myocardial Infarction

**DOI:** 10.14797/mdcvj.1157

**Published:** 2022-12-05

**Authors:** Ryan T. Nguyen, Priyanka Satish, Marvin D. Atkins, Sachin S. Goel

**Affiliations:** 1 Houston Methodist, Houston, Texas, US

**Keywords:** myocardial infarction, ventricular septal rupture

## Abstract

Ventricular septal ruptures (VSR) are a rare but fatal complication of acute myocardial infarctions. We present a patient complaining of new onset heart failure symptoms that were found to be secondary to a new ventricular septal rupture from a recently undiagnosed anterior myocardial infarction. The patient underwent successful VSR patch repair with eventual recovery. This case highlights the importance of early diagnosis of VSR and reviews management options and appropriate timing for intervention.

## Background

Ventricular septal rupture (VSR) is a rare but potentially fatal complication of acute myocardial infarction (MI).^1^ Prior to the reperfusion era, incidence of post-MI VSR was 1% to 3%. However, with the introduction of reperfusion therapy, the incidence of post-MI VSR decreased to 0.2%.^1^ Despite advancements in therapy, post-MI VSR still carries an extremely high in-hospital mortality rate of 33% to 45% with surgical repair and 90% without surgical intervention.^2^ Studies reveal that early identification and delayed surgical repair may decrease mortality rates.^3^ We present a patient with an MI of unknown duration complicated by VSR with signs of decompensated heart failure on admission requiring urgent surgical intervention. This case highlights the echocardiographic and hemodynamic findings and discusses timing of intervention in VSR.

## Case Presentation

A 62-year-old female with a past medical history of hypertension, hyperlipidemia, type 2 diabetes mellitus, and breast cancer (status post chemotherapy and currently on letrozole) presented with progressive shortness of breath and leg swelling. She had an episode of sudden chest pain at home 3 weeks prior to presentation along with a possible cardiac arrest. She received brief cardiopulmonary resuscitation by family with immediate return of spontaneous circulation. Emergency Medical Services was called, although the patient was hemodynamically stable and felt well. She chose not to go to the hospital. She had worsening dyspnea and leg swelling over the next 3 weeks, resulting in her current presentation to our hospital.

Electrocardiogram performed at time of presentation revealed diffuse anterior Q waves. Physical exam was notable for a grade 4/6 holosystolic murmur best heard in the lower sternal border. Transthoracic echocardiogram revealed a severely depressed left ventricular (LV) ejection fraction of 25% to 29% with anteroapical akinesia (Video 1). There was also a VSR with dissection of the septum with a calculated Qp:Qs ratio of 1.70 (Video 2). There was a moderate posterior pericardial effusion and a mild-to-moderate mitral regurgitation.

**Video 1:** Echocardiographic 4-chamber view showing a large area of anteroapical akinesia/dyskinesia, see also at https://youtu.be/abEwJfsJL8Y.

**Video 2:** Echocardiographic 4-chamber view with color Doppler showing an apical ventricular septal defect, see also at https://youtu.be/xjH366swqtY.

**Figure 1:**
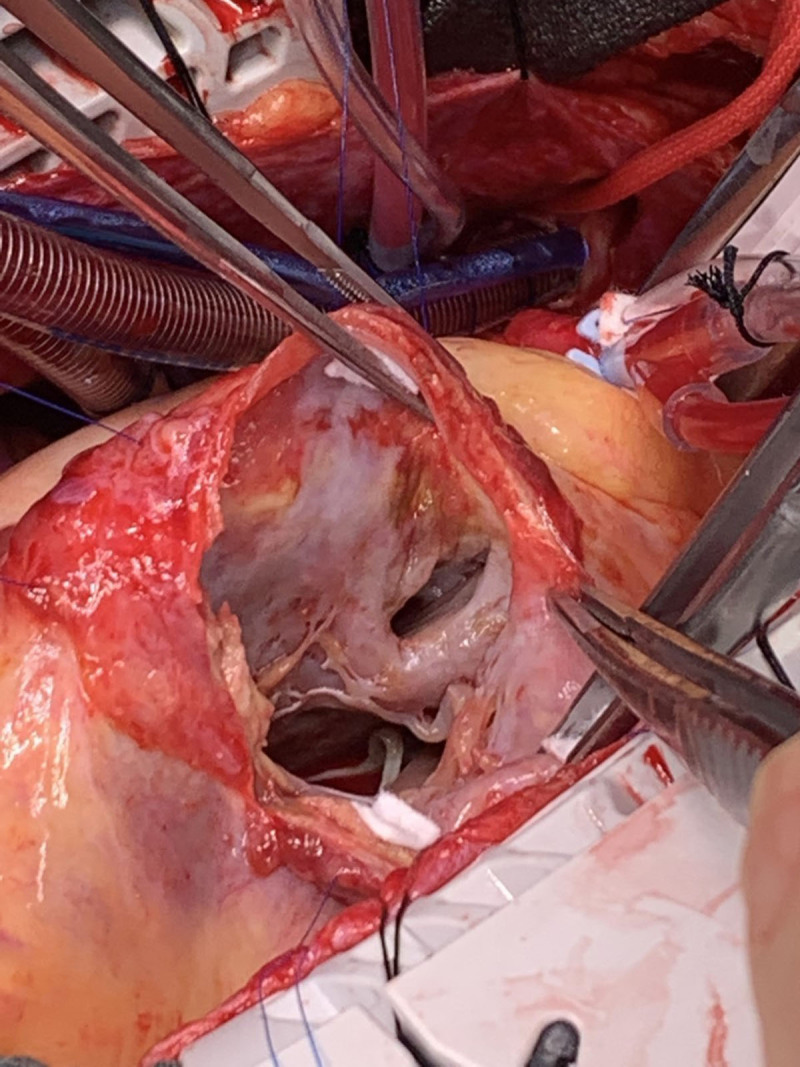
Surgical images of the ventricular septal defect with adhesions from the anterior wall to the pericardial space.

Cardiac catheterization revealed a totally occluded proximal left anterior descending (LAD) coronary artery, mild nonobstructive disease in other epicardial coronaries, elevated biventricular filling pressures, and a significant step-up in oxygen saturation in the right ventricle with a Qp:Qs ratio of 2.06, indicative of significant left-to-right shunt secondary to VSR. An intra-aortic balloon pump (IABP) was placed, and she was taken for emergent surgery. She received a VSR patch repair with resection of the apical aneurysm, mitral valve replacement, and tricuspid ring annuloplasty and was then placed on venoarterial extra corporeal membrane oxygenation (ECMO) (Figure 1). A day later, the ECMO was decannulated, and the IABP was switched to an Impella heart pump (Abiomed). The Impella was removed 5 days later after chest washout and closure. The patient fortunately remained stable and transitioned to a long-term acute care hospital for further recovery.

## Discussion

A VSR is a known complication of MI, with 70% of VSRs occurring in an anterior wall distribution and 29% in the inferior/lateral wall.^4^ The culprit vessel is the LAD in 64% of patients and the right coronary artery in 28% of patients.^2^ Lack of both myocardial preconditioning and collateral circulation increases the likelihood of septal rupture from an acute LAD occlusion.^1^ VSR typically occurs within 1 week after the initial infarction without reperfusion.^1^,^2^,^5^ With reperfusion (either thrombolysis or percutaneous coronary intervention), the median time from infarction to rupture is around 24 to 48 hours.^1^,^2^ Although thrombolytic therapy may decrease the size of the infarct, it can also result in hemorrhage into the myocardium, which can accelerate the onset of the rupture.^1^

Echocardiography with Doppler is a reliable diagnostic modality in identifying VSR, with sensitivity and specificity as high as 100%.^1^,^6^ A Doppler study is also valuable in determining the size and location of the defect and evaluating ventricular function.^1^ VSR associated with anterior infarcts are more likely to be apical simple defects as opposed to inferior infarcts that are complex and associated with posterobasal aneurysms.^7^ Presence of an abnormal Doppler flow signal across the septum into the right ventricle is a characteristic finding of VSR on echocardiography. Right heart catherization (RHC) is a valuable diagnostic tool to determine the degree of interventricular shunting and will reveal a step-up in oxygen saturation in the right ventricle from the right atrium.^1^,^7^ An increase of greater than 10% in the right ventricle is diagnostic of a left-to-right shunt at the ventricular level. Other RHC findings of VSR include mild-to-moderate elevations in right atrial, right ventricle, pulmonary artery, and wedge pressures.^7^

Cardiac surgery with patch repair remains the mainstay treatment for VSR.^3^ Although initially preferred for patients who are poor surgical candidates, percutaneous closure is becoming a more viable and less invasive treatment option for VSR as a definitive strategy or bridge to surgery.^4^,^5^ The timing of surgical or percutaneous intervention remains debatable, with studies suggesting that delaying intervention results in lower mortality rates.^3^,^5^,^8^ Patients who underwent surgery within 7 days of VSR onset had a 54.1% mortality compared with 18.4% mortality if delayed after 7 days.^5^ Similarly in percutaneous closure, mortality was reduced to 21% in those who underwent intervention after the acute phase (> 2 weeks after diagnosis).^8^ The differences in mortality rates between early and delayed surgical intervention may be explained by poor wound healing from weak, friable myocardium in the acute phase and survivor bias from hemodynamically unstable patients requiring emergent surgery.^5^,^8^,^9^ If electing to delay surgical intervention, the main objective of VSR management is to reduce afterload to increase LV stroke volume.^5^ In patients with hemodynamic compromise or multiorgan failure, ECMO can be used with other percutaneous ventricular devices such as Impella or IABP to unload the LV and provide full hemodynamic support.^3^ Postoperatively, these devices can be used temporarily as an adjunct to decompress the LV and support cardiac output.^3^

Our patient presented with acute decompensated heart failure with concern for progression to cardiogenic shock, necessitating IABP placement and emergent cardiac surgery followed by Impella and ECMO support postoperatively. Our patient likely had an anterior MI 3 weeks prior to presentation followed by development of VSR. Although cardiac surgery was emergent on admission, the patient underwent delayed surgery given her late presentation to the hospital. Fortunately, she tolerated the surgery well and is recovering without complications.

## Conclusion

Despite advancements in reperfusion therapy and management of myocardial infarctions, VSR still remains a rare complication that should be considered in a patient presenting with worsening heart failure symptoms and a new holosystolic murmur. Echocardiography often is sufficient in confirming the diagnosis, with right heart catherization being a useful adjunct. Cardiac surgery remains the treatment of choice for VSR, with percutaneous closure becoming a less-invasive alternative option. Although it may be ideal to delay intervention to allow for myocardial healing and better success with patch repair, the timing of intervention varies by case and is dependent on hemodynamic status.
